# Modeling Long-term Vaccination Strategies With MenAfriVac in the African Meningitis Belt

**DOI:** 10.1093/cid/civ508

**Published:** 2015-11-09

**Authors:** Andromachi Karachaliou, Andrew J. K. Conlan, Marie-Pierre Preziosi, Caroline L. Trotter

**Affiliations:** 1 Disease Dynamics Unit, Department of Veterinary Medicine, University of Cambridge, United Kingdom; 2 Meningitis Vaccine Project, PATH, Ferney-Voltaire, France; 3 Meningitis Vaccine Project, Department of Immunization, Vaccines and Biologicals, World Health Organization, Geneva, Switzerland

**Keywords:** meningitis, vaccine, Africa, mathematical modeling

## Abstract

***Background.*** The introduction of MenAfriVac in campaigns targeting people aged 1–29 years across the African meningitis belt has successfully reduced meningitis incidence and carriage due to *Neisseria meningitidis* group A (MenA). It is important to consider how best to sustain population protection in the long term.

***Methods.*** We created a mathematical model of MenA transmission and disease to investigate the potential impact of a range of immunization strategies. The model is age structured; includes classes of susceptible, carrier, ill, and immune people (who may be vaccinated or unvaccinated); and incorporates seasonal transmission and a stochastic forcing term that models between year variation in rates of transmission. Model parameters were primarily derived from African sources. The model can describe the typical annual incidence of meningitis in the prevaccine era, with irregular epidemics of varying size. Parameter and structural uncertainty were explored in sensitivity analyses.

***Results.*** Following MenAfriVac introduction at high uptake, the model predicts excellent short-term disease control. With no subsequent immunization, strong resurgences in disease incidence were predicted after approximately 15 years (assuming 10 years’ average vaccine protection). Routine immunization at 9 months of age resulted in lower average annual incidence than regular mass campaigns of 1- to 4-year-olds, provided coverage was above approximately 60%. The strategy with the lowest overall average annual incidence and longest time to resurgence was achieved using a combination strategy of introduction into the Expanded Programme on Immunization at 9 months, 5 years after the initial mass campaigns, with a catch-up targeting unvaccinated 1- to 4-year-olds.

***Conclusions.*** These results can be used to inform policy recommendations for long-term vaccination strategies with MenAfriVac.

The African meningitis belt suffers from frequent large epidemics of meningococcal meningitis. A novel vaccine against *Neisseria meningitidis* group A (MenA), the major cause of epidemic meningitis, was developed through the Meningitis Vaccine Project (MVP), manufactured by the Serum Institute of India, Ltd [1]. The vaccine, known as MenAfriVac, was first introduced into Burkina Faso, Mali, and Niger in 2010 in mass immunization campaigns targeting 1- to 29-year-olds. MenAfriVac continues to be rolled out across the region, and >217 million individuals have been immunized to date. These campaigns have been remarkably successful in the short term in reducing the incidence of meningitis and the prevalence of MenA carriage, as shown in Burkina Faso [2, 3] and Chad [4]. To ensure that this success continues, long-term immunization strategies are required to maintain population protection.

Computational models have become an important tool for vaccine policy makers. By simulating the impact of a vaccine in silico, a wide range of vaccine strategies can be explored and the sensitivity of their predicted impact to structural and parameter uncertainty can be understood. Transmission dynamic models are essential to quantify both the direct and indirect (herd protection) effects of vaccination programs. For meningococcal infection, most transmission occurs between asymptomatic carriers, so any model attempting to capture the transmission dynamics of meningococci must essentially include the carrier state. This is especially relevant when considering the impact of MenAfriVac, given the evidence that MenA carriage is much reduced following MenAfriVac introduction [2, 4]. This is likely to give rise to large indirect vaccine effects, as seen with other conjugate vaccines [5]. Other key features of the epidemiology of MenA in the African meningitis belt must also be incorporated, which include the periodic but irregular nature of epidemics of varying size; the seasonality of meningitis with epidemics occurring in the dry season and dying out with the onset of the rains [6]; and the variation in disease risk [7] and carriage prevalence [8] by age.

A range of transmission models for meningococcal infection has been developed [9–11]. Only 2 have specifically examined MenA in the African meningitis belt. Irving et al [12] explored the potential mechanisms underlying the striking epidemiology in this region, showing that the complex and irregular timing of epidemics could be explained by the interaction of temporary immunity conferred by carriage of the bacteria together with seasonal changes in the transmissibility of infection. Tartof et al [13] used a transmission model to investigate different strategies using MenAfriVac.

Here we extend the transmission models of Irving et al [12] by addressing some of the limitations (such as the lack of age structure and wide parameter space considered), and incorporating vaccination. We utilize recently available MenA/MenAfriVac specific parameters and apply the model to investigate appropriate policy options for the sustained use of MenAfriVac.

## METHODS

### Model Structure

**Figure 1.**
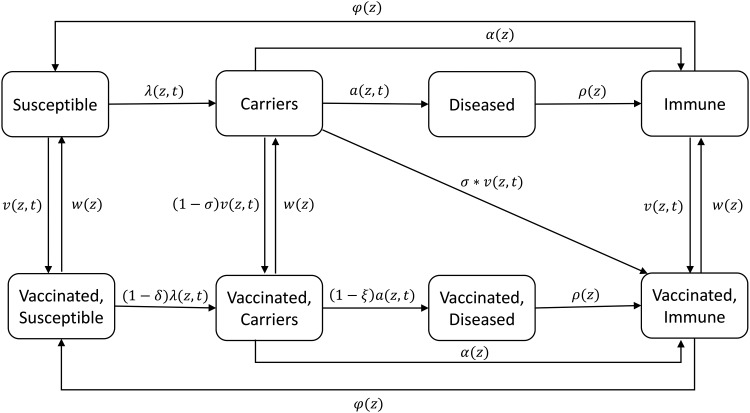
Diagram of the model for *Neisseria meningitidis* group A transmission and disease. Each compartment is divided into distinct age classes (not shown). See Table 1 for definition of parameters and Supplementary Material for the full model structure.

We developed a compartmental model that divides the population into the following states: (1) susceptible, (2) carrier of MenA, (3) disease due to MenA, and (4) recovered and immune, based on our previous investigations of simple deterministic models [12], and in vaccinated populations a mirror of these 4 states (Figure 1). The population is further structured by age into 19 age groups: 0 to <3 months, 3 to <9 months, 9 to <12 months, 1–4 years, 5–9 years, and 5-year age groups to age 80 years subsequently, with continuous aging between groups (rates of aging from one age group to another are given in Supplementary Table 1). The proportion of the population that is in each age group does not change over time.

Vaccination was implemented in different ways according to the strategy used (Table 1). For mass vaccination campaigns, we assumed that immunization occurred as a discrete event at one point in time, whereas routine immunization was implemented continuously as individuals reached the target age for the Expanded Programme on Immunization (EPI). The narrow age groups in <1-year-olds allowed routine vaccination to be implemented at different ages.

**Table 1.** Vaccination Strategies Considered

Vaccine Strategy	Introduction	Long-term
A. Initial campaign only	Mass immunization of 1- to 29-year-olds	Nothing
B. Periodic campaigns	Mass immunization of 1- to 29-year-olds	Periodic mass immunization of 1- to 4-year-olds
C. Routine EPI single dose	Mass immunization of 1- to 29-year-olds	Routine EPI at 9 mo, 5 y after introduction
D. Combination	Mass immunization of 1- to 29-year-olds	Routine EPI at 9 mo, 5 y after introduction, plus catch-up for 1- to 4-year-olds

Abbreviation: EPI, Expanded Programme on Immunization.

An important feature of the meningitis belt is the prominent seasonality [6] of disease, which we implemented through seasonal forcing of the transmission and invasion rates using a sinusoidal function [12]. The baseline transmission rate was varied stochastically drawing from a uniform distribution between 0.8 and 1.2 (ie, ±20%) each year to reflect between-year variation in transmission due to climatic [14] or other external variability. To examine the sensitivity of results to this model structure, we introduced stochasticity in an alternative way, with weekly variation in transmission rates, drawn from the same uniform distribution (0.8–1.2). This “noisy” model used a method similar to the stochastic mechanism used by Tartof et al [13], but with the stochastic term drawn from a narrower range.

Full details of the model structure are given in the Supplementary Material, section A.

### Model Parameters

Model parameters (Table 2) were based on the available literature, and African data wherever possible. Demographic parameters were based on Burkina Faso, a country at the heart of the meningitis belt. Different “who acquires infection from whom” (WAIFW) matrices were used and compared. In the absence of empirical data on population contact patterns, we used evidence on the age distribution of carriers during an MenA epidemic to inform these matrices [20]. The WAIFW matrix used is shown in Supplementary Figure 1; contacts are more intense between individuals in the same age group and particularly so for older children and young adults. It was necessary to estimate values for some parameters where direct evidence was lacking. In exploring the parameter space, it was apparent that there was a strong co-linear relationship between the transmission rate and duration of colonization. Direct estimation of model parameters is complicated by the intractability of the likelihood function for this model and the limitations of available incidence data. As a first exploration of model behavior to guide inference, we found a number of different plausible combinations of parameter values for the transmission rate and duration of natural immunity, which were able to produce realistic results when used in our model and defined a possible range for the unknown parameters.

**Table 2.** Model Parameters

Parameter	Parameter Name	Value	Unit	Comment [Reference]
Mortality rate	d	Age-specific	Years^−1^	Census reports (Supplementary Table 2)
Recovery rate from disease	ρ	52	Years^−1^	Disease lasts about a week [15]
Rate of loss of carriage	α	12	Years^−1^	Only 1 study identified, suggesting 1-mo duration of MenA [16]
Transmission rate	β_0_	10.5	…	Estimate
Rate at which carriers fall ill	a	Age-specific	Years^−1^	Systematic review of case: carrier ratios [17], age-specific parameters estimated (Supplementary Table 3)
Rate of loss of immunity	φ	0.0839	Years^−1^	Estimate, based on previous findings [12]
Seasonal forcing of transmission rate	ε_β_	0.6	…	Estimate, based on previous findings [12]
Seasonal forcing of invasion rate	ε_a_	0.6	…	Seasonality in invasion rate based on published systematic review [17]
Annual growth rate	q	0.0309	Years^−1^	Census reports
Rate of progression between age groups	K	Age-specific	Years^−1^	Estimated using mortality rates and annual population growth rate (Supplementary Table 2)
Vaccine efficacy against carriage	δ	0.6–0.9	Proportion	Range explored, 0.9 from [4]
Vaccine efficacy against disease	ξ	0.6–0.9	Proportion	Range explored, 0.9 from [4]
Carriage clearage upon vaccination	σ	0.9	Proportion	Unknown, effect explored in sensitivity analysis
Waning of vaccine protection	w	0.1	Years^−1^	Consistent with findings from unpublished MVP trials. Varied in sensitivity analysis
Vaccination coverage for initial mass campaign	v_A_	0.95	Proportion	Coverage surveys [18, 19]
Vaccination coverage for additional mass campaigns	v_B_	0.6–0.8	Proportion	Unknown, range explored. 80% used in base case
Vaccination coverage for EPI	v_C_	0.5–0.8	Proportion	Range taken from typical EPI coverage in meningitis belt countries. 80% used in base case

Abbreviations: EPI, Expanded Programme on Immunization; MenA, *Neisseria meningitidis* group A; MVP, Meningitis Vaccine Project.

### Model Implementation

The model was coded and run using the R package version 3.1.0, using the package deSolve to perform the numerical integration of differential equations. The time step was 1 day. For each simulation, we ran the model for a 20-year burn-in period before implementing the initial mass vaccination campaign in year 0. The model was then run for a further 40 years; all results are reported for this 40-year period. For each vaccination strategy, the average of 300 simulations was taken; this was based on a comparison of between 100 and 500 simulations that showed very small marginal differences between 300 and 500 simulations.

### Vaccination Strategies

We considered a range of long-term vaccination strategies and compared these to a scenario without any vaccination and with only an initial mass vaccination campaign of 1- to 29-year-olds (Table 1). We also investigated the sensitivity of the results to changes in the age at EPI immunization and the coverage achieved for EPI immunization at 9 months.

## RESULTS

### Base Case

**Figure 2.**
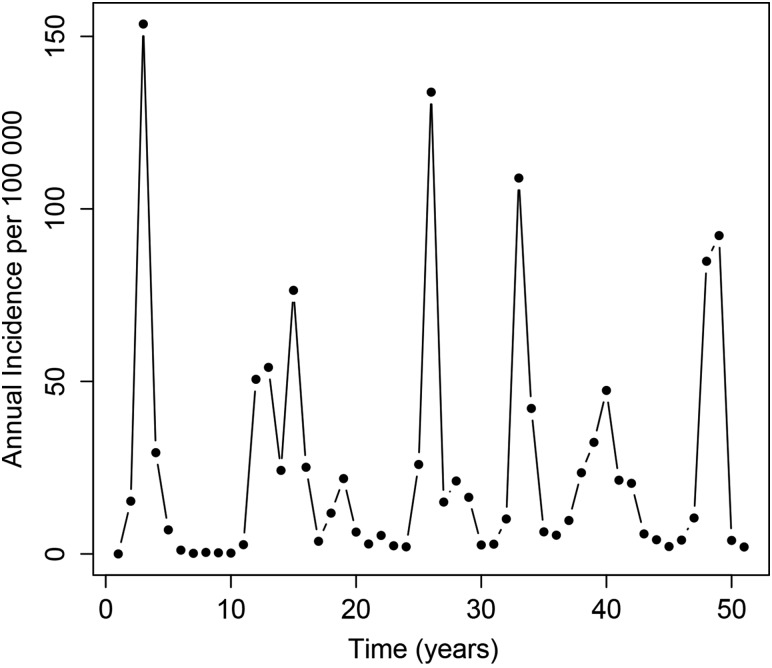
A typical run of the *Neisseria meningitidis* group A transmission model.

In the absence of preventive vaccination, the model was able to capture the distinctive epidemiology of meningococcal infection in the meningitis belt. A typical model run, with irregular epidemics of varying size, is shown in Figure 2.

**Figure 3.**
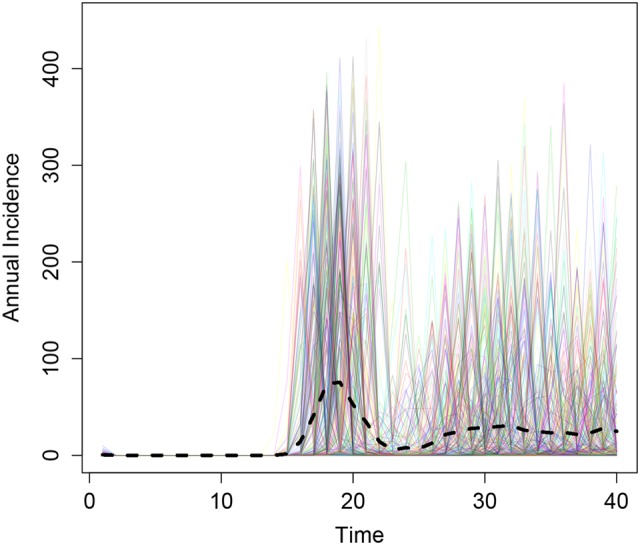
Results from 300 simulations of the initial mass immunization of 1- to 29-year-olds (implemented in year 0). The black dashed line depicts the mean annual incidence.

Following initial mass vaccination of 1- to 29-year-olds, the model predicted a resurgence in disease after approximately 15 years, assuming an average of 10 years of vaccine protection (Figure 3).

**Figure 4.**
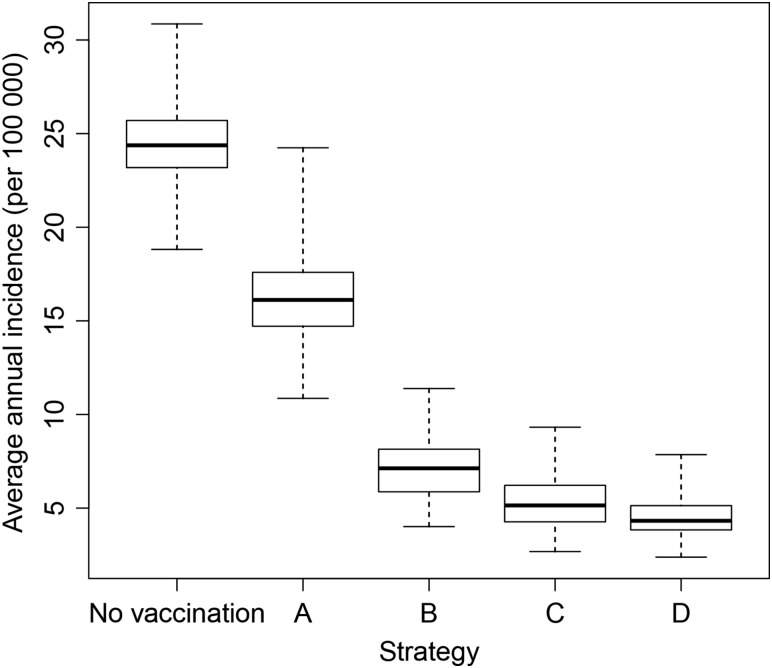
Box plot to show the median, interquartile range, and full range of the predicted annual incidence per 100 000 for different immunization strategies in the 40 years following vaccine introduction from 300 model simulations.

Of the long-term immunization strategies considered, all were effective in maintaining control of disease. There was considerable overlap in the distribution of results (Figure 4), but routine EPI immunization at 9 months of age (strategy C) resulted in lower average annual incidence than regular mass campaigns of 1- to 4-year-olds (strategy B) under base case assumptions. Strategy C was superior to strategy B provided that EPI coverage was above approximately 60% (Table 3). The strategy with the lowest overall average annual incidence and longest time to resurgence was introduction into EPI at 9 months, 5 years after the initial mass campaigns, with a catch-up targeting unvaccinated children aged 1–4 years (strategy D).

**Table 3.** Estimated Average Annual *Neisseria meningitidis* Group A Incidence per 100 000 in the 40 Years Following Vaccine Introduction Under Different Immunization Strategies and Coverage Assumptions

Age, y	No Vaccination	Strategy A	Strategy B				Strategy C	Strategy D
		Mass 1–29 y Only	Mass 1–29 y and EPI at 9 mo at 50% Coverage	Mass 1–29 y and EPI at 9 mo at 60% Coverage	Mass 1–29 y and EPI at 9 mo at 70% Coverage	Mass 1–29 y and EPI at 9 mo at 80% Coverage	Mass 1–29 y and Periodic Mass Campaigns of 1–4 y	Mass 1–29 y Plus EPI at 9 mo and 1–4 y Catch-up
<1	40.32	27.71	14.87	12.49	10.52	8.77	13.52	7.50
1–4	37.38	25.82	10.12	7.94	6.04	4.43	7.15	3.80
5–9	42.54	28.76	13.26	10.92	8.82	6.92	10.00	5.91
10–14	38.61	26.82	14.66	12.57	10.59	8.71	11.48	7.43
15–19	32.14	23.05	14.86	13.24	11.60	9.96	12.10	8.51
20–24	19.18	14.06	9.89	8.99	8.03	7.05	8.26	6.07
25–29	11.20	8.36	6.02	5.51	4.94	4.36	5.10	3.80
≥30	3.18	2.35	1.55	1.39	1.23	1.07	1.30	0.94
All	24.45	17.06	9.01	7.69	6.46	5.31	7.12	4.56

Unless otherwise stated, the coverage attained in the initial mass campaign among 1- to 29-year-olds was 95%, and routine and subsequent catch-up coverage was 80%.

Abbreviation: EPI, Expanded Programme on Immunization.

### Sensitivity Analyses

We investigated the effect of changing some key model parameters and assumptions. In the absence of any long-term immunization (strategy A), assuming a shorter duration of protection resulted in disease incidence increasing more quickly; with 5 years of vaccine protection, the resurgence occurred after around 10 years (not shown).

For strategy C (routine EPI), as expected, as EPI coverage increased, the incidence of disease decreased (Table 3). For every 10% increase, the average annual incidence decreases by approximately 1 case per 100 000 population per year. Also consistent with expectations, disease control was better when vaccine effectiveness was higher (not shown).

We observed only marginal differences by varying the age at which routine MenAfriVac was given. The average disease incidence across all ages decreased as the age at immunization increased from 3 to 9 to 12 months of age. However, there were more cases in infants as the age at routine immunization increased. When averaged across 300 simulations, when MenAfriVac was given routinely at the age of 3 months, the model predicts an average annual incidence of 5.43 cases per 100 000 population per year in all ages and 4.67 cases per 100 000 individuals in infants, compared with the base case of immunization at 9 months (average incidence of 5.31 cases/100 000 across all ages and 8.77 cases/100 000 infants). Immunizing within EPI at 12 months of age results in an average annual incidence of 5.18 cases per 100 000 population, but 10.53 cases per 100 000 in infants.

The model results were insensitive to changes in the assumption of vaccine effectiveness against disease (ξ) when vaccine effectiveness against carriage (δ) was high (90%), because in this situation, carriage acquisitions were rare and so few people were at risk of disease downstream. Because it is unclear whether the vaccine can clear an episode of carriage, we also investigated the sensitivity of the results to changes in clearance upon vaccination. In the base case, we assumed that 90% of the carriers recover immediately after vaccination; when this proportion was changed to 10%, we found that the results were insensitive to the change.

The duration of natural immunity following carriage or disease is not known. In the base case, we assumed on average approximately 12 years’ duration of immunity. When this was lowered to 7 years, keeping other parameters fixed, the incidence of disease under all scenarios was higher. However, the relative ranking of each strategy did not change.

The sensitivity of our results to changes in the model structure were also investigated. The results from the “noisy” model in which the transmission rate varied stochastically each week were very similar to the results presented above.

## DISCUSSION

We developed a model of MenA transmission and disease that was able to describe the epidemiology observed in the African meningitis belt. We simulated the impact of the initial mass vaccination campaigns of 1- to 29-year-olds and predicted a period of very low incidence for at least 10 years, even when assuming a relatively short duration of protection. The indirect effects of the vaccine were clearly important in maintaining this low incidence postintroduction; we assumed a high degree of protection against carriage, consistent with the observed data [2, 4]. Following this honeymoon period, the model predicted a strong resurgence in disease incidence if there was no long-term immunization strategy. Of the long-term strategies we investigated, a combination strategy of routine EPI vaccination after 5 years together with a catch-up campaign targeting children aged 1–4 years who were born after the initial campaigns was the most effective, although there was considerable overlap in the distribution of results for different strategies. Routine EPI alone appeared to be more effective than periodic mass campaigns, unless EPI coverage was low (less than approximately 60%). The model findings, in addition to comprehensive information from clinical trials in children aged <1 year were presented to the World Health Organization's Strategic Advisory Group of Experts (SAGE) on immunization in October 2014 [21].

These findings suggest, first, that it is essential to implement a long-term strategy for the continued use of MenAfriVac. It is not sufficient for the vaccine only to be used in a large one-off campaign, as this may result in catastrophic resurgences in disease 10–20 years after vaccine introduction. All of the long-term strategies considered were effective in maintaining disease control, although for all strategies incidence was predicted to rise over the long term as population immunity from the initial campaigns waned. The inclusion of MenAfriVac into the routine EPI as a single dose at 9 months of age has the obvious advantage of using and likely strengthening existing infrastructure. The option to conduct periodic campaigns may, however, provide better disease control for those countries with very poor routine EPI uptake. The combination strategy of introduction into routine EPI with a one-off catch-up campaign targeting those born since the initial campaign was the most effective and also the most equitable option. Indeed, SAGE recommended that countries should adopt such a strategy within 5 years of campaign completion [22].

Our work has several strengths and limitations. Our model structure was based on extensive previous work that used a range of deterministic models, to explore the importance of seasonality and immunity following colonization [12]. As such, we feel we have good understanding of the underlying system dynamics. We extended these models to incorporate age structure and vaccination, and included a stochastic term so that the extent of seasonal forcing varied from year to year, to capture the effect of external forces (including, eg, dust or humidity conditions) [23]. We parameterized the model using appropriate published and unpublished data specific to African populations as far as possible. Some model parameters were unknown, including the transmission rate and duration of natural immunity. Here, we used a variety of methods to estimate a sensible range and feasible parameter combinations, ensuring that the model produced realistic results by comparing the model predictions to evidence on carriage prevalence by age, disease incidence by age, total annual incidence, seasonality, and periodicity. Further investigation of formal fitting methods such as Approximate Bayesian Computation is warranted [24], and more information on a range of parameters would be desirable, including age-specific contact patterns. Quantifying the duration of natural immunity following infection is particularly difficult; estimation is hampered by codependence with other parameters, and empirical measurement is problematic, not least because of the lack of an absolute correlate of protection [25]. We performed sensitivity analyses to investigate parameter uncertainty and showed that our findings were robust.

Our conclusions are different from another model of MenAfriVac, which found that mass campaigns were superior to routine EPI. This is probably largely because the duration of protection assumed by Tartof et al was much greater (essentially lifelong) for children immunized in campaigns than through EPI [13], whereas we assumed that protection in 1- to 4-year-olds would be similar to those immunized at the age of 9 months, based on recent data from the MVP's MenAfriVac trials. Tartof et al also used a different model structure, a larger time step, noncontinuous aging, a smaller number of simulations, and a higher frequency (weekly) and amplitude (0–0.75) of stochastic forcing. We chose a more parsimonious model structure that did not consider variable levels of protection against colonization and disease, as there was little evidence to inform such a structure and its parameterization. We explored the effect of other structural changes in our model, including the implementation of stochasticity as weekly variation in transmission rates, but this had minor effects on the model predictions and did not change our conclusions on the relative merits of each immunization strategy.

Following its introduction in 2010, MenAfriVac has been remarkably successful in controlling MenA disease. This success will not be maintained without a long-term immunization strategy. The early adopting countries will need to consider imminently how best to sustain population protection against MenA, and findings from mathematical models such as this can lend further support to decision makers at both the country level and internationally.

## Supplementary Data

Supplementary materials are available at *Clinical Infectious Diseases* online (http://cid.oxfordjournals.org). Supplementary materials consist of data provided by the author that are published to benefit the reader. The posted materials are not copyedited. The contents of all supplementary data are the sole responsibility of the authors. Questions or messages regarding errors should be addressed to the author.

Supplementary Data
