# Supplementary material for: Modeling Long-term Vaccination Strategies With MenAfriVac in the African Meningitis Belt
Source: Clin Infect Dis. 2015 Nov 9;61(Suppl 5):S594–600. doi: 10.1093/cid/civ508 (PMC4639487; doi:10.1093/cid/civ508)
Supplement: Supplementary Data [file supp_61_suppl-5_S594__index.html]

Supplementary Data 

# Modeling Long-term Vaccination Strategies With MenAfriVac in the African Meningitis Belt

## Supplementary Data

Supplementary Data

- Supplementary Data - Docx file
