# Supplementary material for: Modeling Long-term Vaccination Strategies With MenAfriVac in the African Meningitis Belt
Source: Clin Infect Dis. 2015 Nov 9;61(Suppl 5):S594–600. doi: 10.1093/cid/civ508 (PMC4639487; doi:10.1093/cid/civ508)
Supplement: Supplementary Data [file supp_civ508_civ508supp.docx]

**SUPPLEMENTARY MATERIAL**

**A. Detailed model structure**

The following differential equations define the rates at which individuals move between different states:

$$\frac{dS_{1}}{dt}=\left( K_{1}+d_{1}+q \right)P_{1}+\varphi R_{1}-(\lambda_{1}+d_{1}+K_{1}+q)S_{1}$$

$$\frac{dS_{j}}{dt}=K_{j-1}S_{j-1}+\varphi R_{j}-\left( \lambda_{j}+d_{j}+K_{j}+q \right)S_{j}, 2\leq j\leq19$$

$$\frac{dC_{1}}{dt}=\lambda_{1}S_{1}-(a_{1}+\alpha+d_{1}+q+K_{1})C_{1}$$

$$\frac{dC_{j}}{dt}=K_{j-1}C_{j-1}+\lambda_{j}S_{j}-\left( a_{j}+\alpha+d_{j}+q+K_{j} \right)C_{j}, 2\leq j\leq19$$

$$\frac{dI_{1}}{dt}=a_{1}C_{1}-(\rho+d_{1}+q+K_{1})I_{1}$$

$$\frac{dI_{j}}{dt}=K_{j-1}I_{j-1}+a_{j}C_{j}-\left( \rho+d_{j}+q+K_{j} \right)I_{j}, 2\leq j\leq19$$

$$\frac{dR_{1}}{dt}=\rho I_{1}+\alpha C_{1}-\left( \varphi+d_{1}+q+K_{1} \right)R_{1}$$

$$\frac{dR_{j}}{dt}=K_{j-1}R_{j-1}+\rho I_{j}+\alpha C_{j}-\left( \varphi+d_{j}+q+K_{j} \right)R_{j}, 2\leq j\leq19$$

Where the states are Susceptible (S), Carrier (C), Ill (I), Recovered and immune (R). The subscript j refers to age group.

The model parameters are force of infection (λ_j_); rate of progression between age groups (K_j_); age-dependent death rate (d_j_); annual population growth rate (q); rate of loss of immunity (𝜑); rate of loss of carriage (𝛼); rate at which carriers fall ill ($a$); recovery rate from disease (𝜌); seasonal forcing of transmission rate (𝜀_𝛽_); seasonal forcing of invasion rate (𝜀_a_).

The force of infection, seasonally forced transmission rate and seasonally forced transmission rate are further defined below.

$$\lambda_{j}=\theta\sum_{k=1}^{n} \beta(z_{j},z_{k})(I_{k}+C_{k})$$

$$\beta\left( z_{j},z_{k} \right)= \beta_{0}\left( z_{j},z_{k} \right)(1+\varepsilon_{\beta}\cos(2\pi t))$$

$$a_{j}= a_{0j}(1+\varepsilon_{a}\cos(2\pi t))$$

At time t = t_v_, a mass vaccination of 1-29 year olds is implemented, whereby

$$SV_{j}=pS_{j}, 4\leq j\leq9$$

$$CV_{j}=\left( 1-\sigma\right)pC_{j}, 4\leq j\leq9$$

$$IV_{j}=pI_{j}, 4\leq j\leq9$$

$$RV_{j}=pR_{j}+\sigma pC_{j}, 4\leq j\leq9$$

$$S_{j}=S_{j}-pS_{j}, 4\leq j\leq9$$

$$C_{j}={C_{j}-pC}_{j}, 4\leq j\leq9$$

$$I_{j}={I_{j}-pI}_{j}, 4\leq j\leq9$$

$$R_{j}=R_{j}-pR_{j}, 4\leq j\leq9$$

For j=2,..,19

and m=5,6,…,19, where m=age group of vaccinated individuals

$$\frac{dS_{1}}{dt}=\left( K_{1}+d_{1}+q \right)P_{1}+\varphi R_{1}-(\lambda_{1}+d_{1}+K_{1}+q)S_{1}$$

$$\frac{dS_{j}}{dt}=K_{j-1}S_{j-1}+\varphi R_{j}-\left( \lambda_{j}+d_{j}+K_{j}+q \right)S_{j}+w{SV}_{j}$$

$$\frac{d{SV}_{4}}{dt}=\varphi{RV}_{4}-\left( \left( 1-\delta\right)\lambda_{4}+d_{4}+K_{4}+q+w \right){SV}_{4}$$

$$\frac{d{SV}_{m}}{dt}=K_{m-1}{SV}_{m-1}+\varphi{RV}_{m}-\left( \left( 1-\delta\right)\lambda_{m}+d_{m}+K_{m}+q+w \right){SV}_{m}$$

$$\frac{dC_{1}}{dt}=\lambda_{1}S_{1}-(a_{1}+\alpha+d_{1}+q+K_{1})C_{1}$$

$$\frac{dC_{j}}{dt}=K_{j-1}C_{j-1}+\lambda_{j}S_{j}-\left( a_{j}+\alpha+d_{j}+q+K_{j} \right)C_{j}+w{CV}_{j}$$

$$\frac{d{CV}_{4}}{dt}=\left( 1-\delta\right)\lambda_{4}{SV}_{4}-\left( \left( 1-\xi\right)a_{4}+\alpha+d_{4}+q+K_{4}+w \right){CV}_{4}$$

$$\frac{d{CV}_{m}}{dt}=K_{m-1}{CV}_{m-1}+\left( 1-\delta\right)\lambda_{m}{SV}_{m}-\left( \left( 1-\xi\right)a_{m}+\alpha+d_{m}+q+K_{m}+w \right){CV}_{m}$$

$$\frac{dI_{1}}{dt}=a_{1}C_{1}-(\rho+d_{1}+q+K_{1})I_{1}$$

$$\frac{dI_{j}}{dt}=K_{j-1}I_{j-1}+a_{j}C_{j}-\left( \rho+d_{j}+q+K_{j} \right)I_{j}$$

$$\frac{d{IV}_{4}}{dt}=\left( 1-\xi\right)a_{4}{CV}_{4}-\left( \rho+d_{4}+q+K_{4} \right){IV}_{4}$$

$$\frac{d{IV}_{m}}{dt}=K_{m-1}{IV}_{m-1}+\left( 1-\xi\right)a_{m}{CV}_{m}-\left( \rho+d_{m}+q+K_{m} \right){IV}_{m}$$

$$\frac{dR_{1}}{dt}=\rho I_{1}+\alpha C_{1}-\left( \varphi+d_{1}+q+K_{1} \right)R_{1}$$

$$\frac{dR_{j}}{dt}=K_{j-1}R_{j-1}+\rho I_{j}+\alpha C_{j}-\left( \varphi+d_{j}+q+K_{j} \right)R_{j}+w{RV}_{j}$$

$$\frac{d{RV}_{4}}{dt}=\rho{IV}_{4}+\alpha{CV}_{4}-\left( \varphi+d_{4}+q+K_{4}+w \right){RV}_{4}$$

$$\frac{d{RV}_{m}}{dt}=K_{m-1}{RV}_{j-1}+\rho{IV}_{m}+\alpha{CV}_{m}-\left( \varphi+d_{m}+q+K_{m}+w \right){RV}_{m}$$

Where additional compartments represent vaccinated individuals who are Susceptible (SV), Carrier (CV), Ill (IV), Recovered and immune (RV).

Additional parameters are vaccination coverage (p); vaccine efficacy against carriage (𝛿); carriage clearance upon vaccination (𝜎); vaccine efficacy against disease (𝜉); rate at which vaccinated revert to unvaccinated compartment (w). Routine EPI vaccination is implemented at birth (j=1), age 3 months (j=2), 9 months (j=3) or 12months (j=4) using a similar system as described in the equations above for mass campaigns, but vaccination occurs continuously rather than as a discrete event. Mass vaccination of 1-4 year olds subsequent to the initial campaigns is implemented as above, but in just one age class.

The force of infection is thus defined as:

$$\lambda_{j}=\theta\sum_{k=1}^{n} \beta(z_{j},z_{k})(I_{k}+C_{k}+{IV}_{k}+{CV}_{k})$$

And the transmission and invasion rates as:

$$\beta\left( z_{j},z_{k} \right)= \beta_{0}\left( z_{j},z_{k} \right)(1+\varepsilon_{\beta}\cos(2\pi t))$$

$$a_{j}= a_{0j}(1+\varepsilon_{a}\cos(2\pi t))$$

**B. WAIFW matrices and model parameters**

Several different ‘who acquires infection from whom’ (WAIFW) matrices were used and compared in preliminary analyses (following Tom Irving’s PhD thesis, University of Bristol 2013). These matrices describe the age-specific contact rates between individuals within and between different age groups. The WAIFW matrix that produced the best visual fit to the observed age-specific prevalence of colonization was an adaptation of the preferential mixing (figure S1).

*Figure S1: Adapted WAIFW matrix.*

*
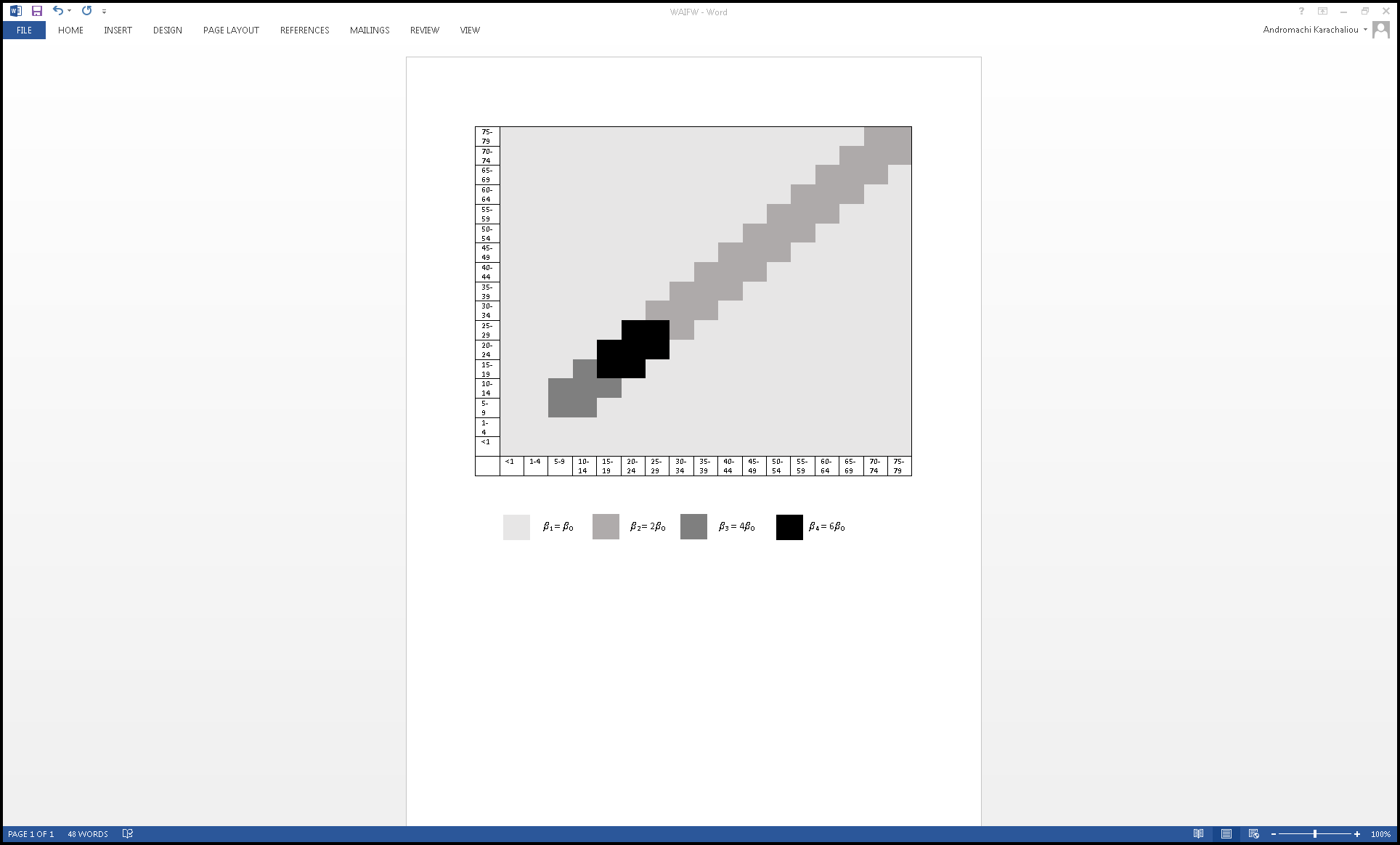
*

**Supplementary tables**

*Table S1: Parameter values used in our model, where K_j_ is the rate of progression between age groups*

| K_1_  3.9415673 | K_2_  1.9418546 | K_3_  3.9415673 | K_4_  0.22615 | K_5_  0.183166 | K_6­_  0.184199 | K_7_  0.183713 |
| --- | --- | --- | --- | --- | --- | --- |
| K_8_  0.183208 | K_9_  0.182991 | K_10_  0.182921 | K_11_  0.182582 | K_12_  0.182187 | K_13_  0.18125 |  |
| K_14_  0.179814 | K_15_  0.176913 | K_16_  0.173196 | K_17_  0.165751 | K_18_  0.153113 | K_19_  0 |  |

The transfer rates K_j_ were calculated from

$$K_{j}=\frac{(d_{j}+q)}{\exp\left[ \left( d_{j}+q \right)\left( z_{j}-z_{j-1} \right) \right]-1}$$

Where d_j_ is the age-dependent mortality rate, q is the country’s annual growth rate and z denotes the age.

*Table S2: Parameter values used in our model, where d_j_ is the age-dependent death rate*

| d_1_  0.08654 | d_2_  0.08654 | d_3_  0.08654 | d_4_  0.01842 | d_5_  0.00377 | d_6­_  0.00158 | d_7_  0.00261 |
| --- | --- | --- | --- | --- | --- | --- |
| d_8_  0.00368 | d_9_  0.00414 | d_10_  0.00429 | d_11_  0.00501 | d_12_  0.00585 | d_13_  0.00785 |  |
| d_14_  0.01093 | d_15_  0.0172 | d_16_  0.02534 | d_17_  0.04202 | d_18_  0.07159 | d_19_  0.2 |  |

*Table S3: Parameter values of a that are typically unknown for the 19-age class structured model that were used in simulations. These values give good agreement with observed data.*

| a_01_  0.15535 | a_02_  0.15535 | a_03_  0.15535 | a_04_  0.1434 | a_05_  0.0956 | a_06­_  0.08365 | a_07_  0.0717 |
| --- | --- | --- | --- | --- | --- | --- |
| a_08_  0.0478 | a_09_  0.03585 | a_010_  0.0239 | a_011_  0.0239 | a­_012_  0.01195 | a_013_  0.01195 |  |
| a_014_  0.01195 | a_015_  0.01195 | a_016_  0.00239 | a_017_  0.00239 | a_018_  0.00239 | a_019_  0.00239 |  |
